# Obituary: Robert Jennings

**Published:** 1893-10

**Authors:** 


### OBITUARY.

Professor Robert Jennings, V.S., died at Detroit, Mich., January 5th, 1893, aged 78 years. His name will always be closely identified with the early history of veterinary education in America. He acquired a practical knowledge of veterinary science by studying sometime with a veterinary practitioner, and matriculated in the Pennsylvania Medical College, but never graduated; then delivered a series of veterinary lectures to a number of medical students from several medical colleges in Philadelphia during the winter months of 1846 to 1850, and finally founded the Veterinary College of Philadelphia, which was chartered April 15th, 1852. This was the pioneer veterinary college of America, and failed to prove a financial success. The faculty disorganized and the college closed its doors March 3d, 1866, and was succeeded by the Pennsylvania College of Veterinary Surgeons, which was chartered April 11th, 1866. The faculty disbanded and the college ceased to exist after 1870. A number of students were educated and received diplomas from these colleges. He introduced the castrating ecraseur in this country in 1852; organized the American Veterinary Association in 1854, and was veterinary lecturer in Ohio Agricultural College duirng 1856-7. A series of books on diseases of domestic animals and horse training was prepared by Dr. Jennings from copious extracts from British authors, and proved very useful to live-stock owners in this country. He corresponded with numerous veterinary practitioners, and issued a circular-letter which resulted in the formation of the United States Veterinary Medical Association June 9th, 1863; and this is a fitting and everlasting monument to his memory. A number of interesting contributions from his pen may be found in the early volumes of THE JOURNAL OF COMPARATIVE MEDICINE AND SURGERY. He was a member of the Board of Censors of the Columbia Veterinary College, and at one time took a very active interest in the advancement of the veterinary profession in this country. His son, Dr. Robert Jennings, Jr., is a veterinary practitioner in Pittsburgh, Pa.

